# 

**Published:** 1865-11

**Authors:** 


### CONTENTS.

ORIGINAL ESSAYS AND COMMUNICATIONS.
Extraction of Teeth without the Lancet.... 441

PROCEEDINGS OF SOCIETIES.
Central Ohio Dental Association.... 446

SELECTIONS.
Teaching.... 447
The Food and the Teeth.... 455
Arsenic.... 478
Annual Meeting of the Merimack Valley Dental Association.... 480
Paralysis of Arm Resulting from a Carious Tooth.... 481
Smoking.... 481
New Test for Arsenic.... 483

Subjects for discussion.—Society of Dental Surgeons of the City of New York.... 482
Subjects for discussion.—Society of Dental Surgeons of the City of Brooklyn.... 482
Subjects of Discussion by the Cincinnati Dental Association.... 486

EDITORIAL.
Personal.... 484
Atlantic & Great Western R. W.... 484
The Cincinnati Dental College Society.... 485
Ohio State Dental Society.... 485
Pecuniary.... 486

